# Supplementary material for: The Anti-Tumor Effect of the Newly Developed LAT1 Inhibitor JPH203 in Colorectal Carcinoma, According to a Comprehensive Analysis
Source: Cancers (Basel). 2023 Feb 22;15(5):1383. doi: 10.3390/cancers15051383 (PMC10000236; doi:10.3390/cancers15051383)
Supplement: Supplementary file 1 [file cancers-15-01383-s001.zip › cancers-2190188-supplementary.pdf]

Cancers

The anti-tumor effect of the newly developed LAT1 inhibitor JPH203 in colorectal carcinoma, according to a comprehensive analysis

Rina Otani, Hidehiko Takigawa, Ryo Yuge, Daisuke Shimizu, Misa Ariyoshi, Ryo Miyamoto, Hiroki Kadota, Yuichi Hiyama, Ryohei Hayashi, Yuji Urabe, Akira Ishikawa, Naohide Oue, Yasuhiko Kitadai, Shiro Oka, Shinji Tanaka

Corresponding Author:

Hidehiko Takigawa, M.D., Ph.D.

Department of Endoscopy, Hiroshima University Hospital, Hiroshima, Japan

Hiroshima University Hospital

1-2-3 Kasumi, Minami-ku, Hiroshima, Hiroshima 734-8553, Japan

Telephone and Fax Number: +81-82-257-5939

Email: hidehiko@hiroshima-u.ac.jp

Table S1. Primers used in this research

Target gene	Direction	Sequence (5'-3')	Product size (bp)
human-GAPDH	Forward	CCACCCATGGCAAATTCC	72
	Reverse	TGATGGGATTTCCATTGATGAC	
human-LAT1	Forward	CCGAGGAGAAGGAAGAGGC	161
	Reverse	GAAGATGCCCGAGCCGATAA	
mouse-GAPDH	Forward	GCCTCGTCCCGTAGACAAAA	207
	Reverse	CCATTCTCGGCCTTGACTGT	
mouse-LAT1	Forward	CCTCAAACGTCAGGTGTCCA	143
	Reverse	TCCCCAGAAAGAAAGTCCAGG	
